# Estimation of the Three Phases by Direct Cost of Care for Non-surviving Patients with Cancer: A National Population-based Patient-level Study

**DOI:** 10.7150/jca.78491

**Published:** 2024-01-01

**Authors:** Tae Mi Youk, Jung Hwa Hong, Byung Kyu Park, Young Min Park, Eun-Cheol Park

**Affiliations:** 1 Research Institute, National Health Insurance Service Ilsan Hospital, Goyang, Republic of Korea.; 2 Department of Statistics, Korea University, Seoul, Republic of Korea.; 3 Division of Gastroenterology, Department of Internal Medicine, National Health Insurance Service Ilsan Hospital, Goyang, Republic of Korea.; 4 Department of Family Medicine, National Health Insurance Service Ilsan Hospital, Goyang, Republic of Korea.; 5 Department of Preventive Medicine and Institute of Health Services Research, Yonsei University College of Medicine, Seoul, Republic of Korea.

**Keywords:** cost of cancer care, cancer deaths, continuous piecewise model, change points

## Abstract

**Background:** Determining the cost structure of medical care from diagnosis to the death of patients with cancer is crucial for establishing budgets to support patients with cancer. The breakdown of the cost estimation in distinct phases of survival is essential for optimizing the allocation of limited funds. Therefore, this study aims to examine the patterns of direct medical costs of cancer care associated with seven major cancer types and estimate cost thresholds to distinguish each phase based on the incurred cost.

**Methods:** In this nationwide, population-based study, we used claims data from the National Health Insurance Service, Korea. Patients newly diagnosed with cancer since 2006 and who died in 2016-2017 were enrolled, and their use of medical services during cancer survival from at least 6 months up to 12 years was observed. The monthly cost exhibited a non-linear function with two unknown thresholds resembling a U-shape; therefore, we fitted three linear segment models. Individual costs were assessed by dividing the survival time into the initial, continuing, and terminal phases by estimated thresholds, and the average medical cost for each phase was calculated.

**Results:** Based on survival durations of 12 years or less, the initial phase occurred within 1.1-4.8 months after diagnosis, while the terminal phase was observed in 1.4-4.7 months before death. The length of these two phases increased with the increased survival time of the patients. Medical costs in these phases ranged from $4067-7431 and $3127-6114 (US dollars), respectively, regardless of the variations in survival time. However, the average costs in the continuing phase were higher for patients with a short survival time.

**Conclusions:** This study highlights the cost dynamics in cancer care through a breakdown of the phases of survival. It suggests that through a more refined definition of the initial and terminal phases, the average cost in these stages increases, indicating the significant implications of the findings for resource allocation and tailored financial support strategies for patients with cancer with varying prognoses.

## Background

Cancer is one of the major causes of death worldwide. In 31 European cities, approximately 3.1 million people were newly diagnosed with cancer and 1.4 million people died of cancer in 2018 1. In the United States, 1.8 million people were newly diagnosed with cancer, and the number of cancer deaths was 0.6 million in 2020 2. Worldwide, 18.1 million new cancers were diagnosed and 9.5 million cancer deaths occurred in 2018. By 2040, the number of new cancer cases per year is estimated to be 29.5 million and the number of cancer-related deaths 16.4 million 3. The age-standardized rate for all cancers in Korea in 2020 was 482.9 per 100,000 people, which is lower than that of the United States or the United Kingdom 4. The relative survival rates of patients with cancer between 2016 and 2020 were 71.5% for 5 years and 67.5% for 10 years, and are continuously improving. However, cancer is still the number one cause of death in Korea in 2020, and the death rate is 160.1 per 100,000 people 5.

The number of patients with cancer is increasing worldwide, and organizing the money they spend while suffering from cancer is an important issue both nationally and personally. Knowing the expenses incurred for the overall treatment, surgery, and examination from diagnosis until death can help estimate future resource requirements and enable precise cost-effectiveness analyses. This knowledge can serve as the foundation of insurance policies for the management of patients with cancer, ensuring that the allocation of funds aligns accurately with their needs. Moreover, setting a precise cut-off by breaking down the disease duration into distinct phases can prevent overestimation or underestimation of cost. Currently, cancer-related burdens are increasing in many developed countries 6. In particular, Korea, which has a nationwide medical insurance system, has an expanding benefit coverage for patients with cancer, where the government covers the majority, and the patients need to pay only 5% of the total amount of medical expenses. Therefore, accurate estimation of the cost of care holds immense sway over policy budgeting.

Direct costs encompass all expenses associated with the use of medical resources, including hospitalization, outpatient services, and pharmaceuticals. Individual costs are high for the examination, initial surgery, and treatment near the onset of cancer, but they tend to stabilize during the treatment process. However, as the patient approaches death, medical costs tend to escalate rapidly 7. The Surveillance, Epidemiology, and End Results (SEER) program shows that for patients with breast cancer over 65 years old, the annual costs one year post-diagnosis are $23,078; decreasing to $2,207 in the interim period; and surging to $62,856 in the last year of life 8. In the case of colorectal cancer, healthcare costs are $36,092 and $41,562 for stages II and IV, respectively, one-year post-diagnosis 9. In the interim period, costs amount to $3,216 and $20,582 for the same stages, while in the last year of life, they are $12,755 and $25,714, respectively. The treatment modality for newly diagnosed patients with lung cancer can be applied differently depending on the disease stage 10. Yearly medical costs in the case of surgery alone, surgery combined with chemo or radiotherapy, chemo or radiotherapy without surgery, and best supportive care stand at $4,359; $7,075; $7,626; and $12,042; respectively, providing valuable insights into the varying financial aspects of cancer care.

Understanding the trajectory of direct medical costs of an individual throughout their cancer survival journey, which follows a non-linear pattern over time, is crucial. Good fitting the proper function and observing the structural change plays an important role in the field of economics and statistics, as it enables the generalization of the cost patterns and serves as a foundational element for predictive modeling. The direct medical cost of care from diagnosis to death in patients with cancer often exhibits a U-shape 7. While previous studies have traditionally defined the initial and terminal phases as the first six months or last year of life after diagnosis, the challenge lies in the arbitrary nature of these timeframes. Hence, it becomes imperative to reevaluate and recalibrate these phases based on empirical data to ensure their alignment with real-world dynamics. The period spanning from cancer diagnosis to death is called cancer survival time and can be divided into three phases: initial, continuing, and terminal. Typically, the initial phase encompasses the first 6 or 12 months post-diagnosis, while the terminal phase encapsulates the last 6 or 12 months of life 7-9,11-17. The interval between the initial and terminal phases is defined as the continuing phase. Therefore, in previous studies exploring cancer costs in accordance with these phases under the same conditions, a prerequisite was set for a minimum cancer survival time of at least one year. However, this criterion often led to the exclusion of patients with rapidly progressing cancers, such as pancreatic cancer, due to their short median survival time. Therefore, it is essential to reconsider the change points that separate the phase when analyzing the cost during the survival period.

The direct cost follows a non-linear function. The increased cost near diagnosis decreases as time passes by, then maintains a monotonic increase for a certain period, and increases rapidly again as the end of life approaches. These serve as benchmarks for distinct cost patterns. By optimizing the choice of these points, such as at 6 or 12 months, can smooth out the average cost within each phase. Therefore, we estimated the unknown change points that can effectively distinguish the phase based on variations in average medical cost, ultimately defining the cost dynamics in each phase. The purpose of this study was to examine the changes in the pattern of direct costs of cancer care during the survival period for each cancer type using data on cancer deaths in Korea. By estimating its unknown change point, we established a phase of care cost. In addition, we determined the scale of cost in each phase.

## Study Population and Methods

### Study population

In this study, claims data from the National Health Insurance Service (NHIS) from Korea were used to analyze the medical cost of cancer deaths. Although NHIS is not a cancer registry, patients with cancer can be accurately identified from claims data due to the specific benefits they receive, which often cover out-of-pocket expenses. Information such as their use of medical services, insurance status, general characteristics, and death dates can also be retrieved from these data.

**Figure 1:**
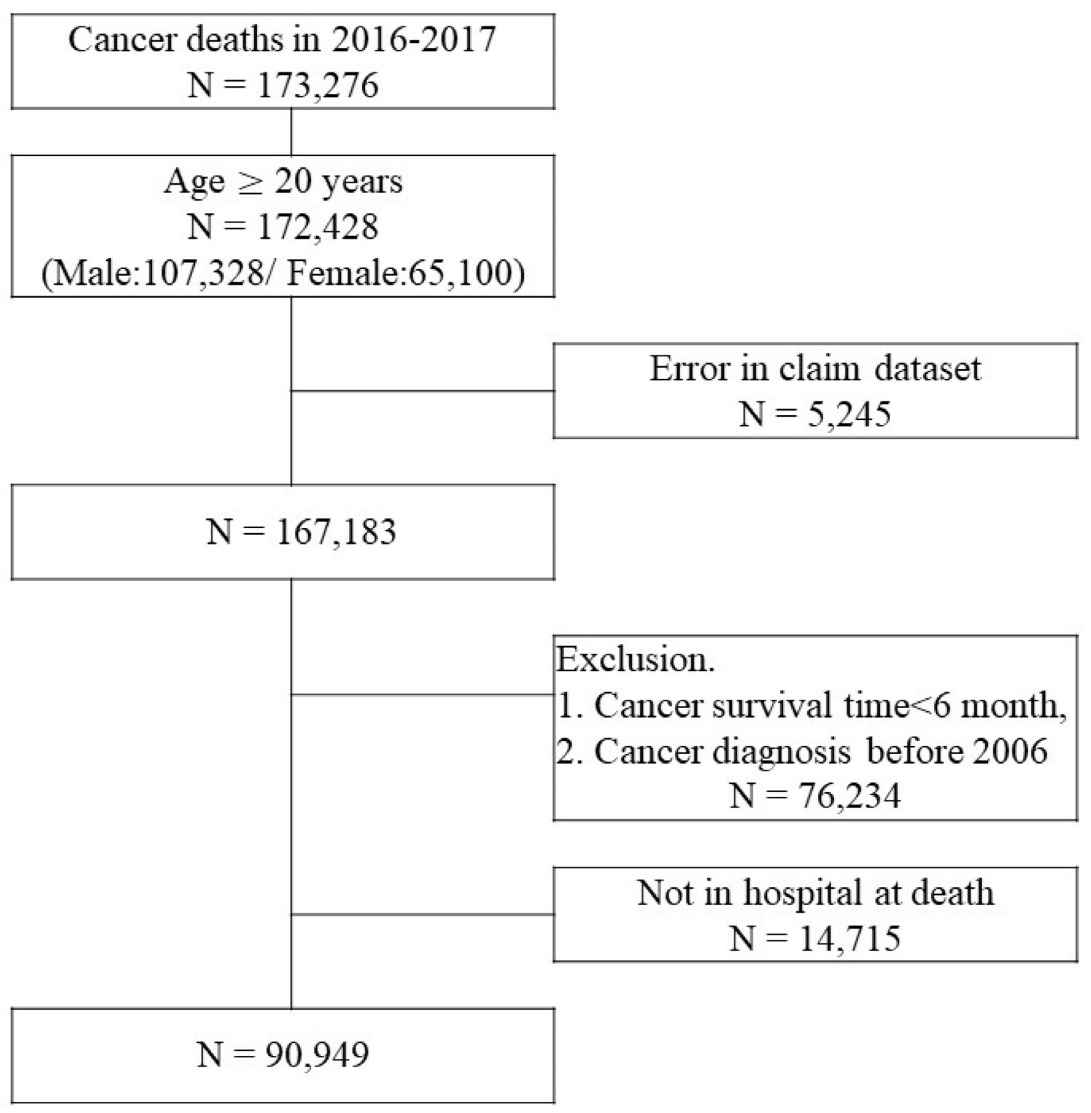
Selection of study subjects.

The total number of cancer deaths in Korea from 2016 to 2017 was 173,276, of which the following cases were excluded from the study subjects (Figure 1): under the age of 20, if there was an error in the claims data, deaths within 180 days of cancer diagnosis, cancer diagnosis before 2006, and not in-hospital death. The final number of study subjects was 90,949. In 2006-2017, approximately 2,490,327 new patients with cancer with average 5-year and 10-year survival rates of 64% and 55.1%, respectively, were reported 11. However, in this study, deaths in a particular year were considered only to reflect similar policies or medical charges.

The type of cancer of each patient was defined based on the International Classification of Diseases 10^th^ revision (ICD-10) codes at the time of death. Specifically, lung cancer was categorized under ICD-10 code C33-34; stomach cancer, C16; colorectal cancer, C18-20; breast cancer, C50; pancreas cancer, C25; liver cancer, C22; and gallbladder and extrahepatic bile cancer, C23-24. These types represent the most prevalent and significant cancers in terms of incidence and deaths in Korea.

### Direct medical cost of cancer care

The statement of use of medical services for patients with cancer collected by NHIS includes the total medical costs executed as well as out-of-pocket expenses. In addition, since the main and sub-diseases are specified and the cancer-related services were distinguished, the direct cost of cancer care in this study did not consider non-cancer-related services. We defined hospitalization costs as the sum of all operations performed during hospitalization, such as surgery, treatment, examination, prescription drugs, hospital room fees, meals, and management fees. The outpatient costs represented all expenses (including prescriptions) incurred while using a medical institution without being hospitalized. Direct medical costs of cancer care followed each individual's hospitalization and outpatient costs from cancer diagnosis to death.

**Figure 2:**
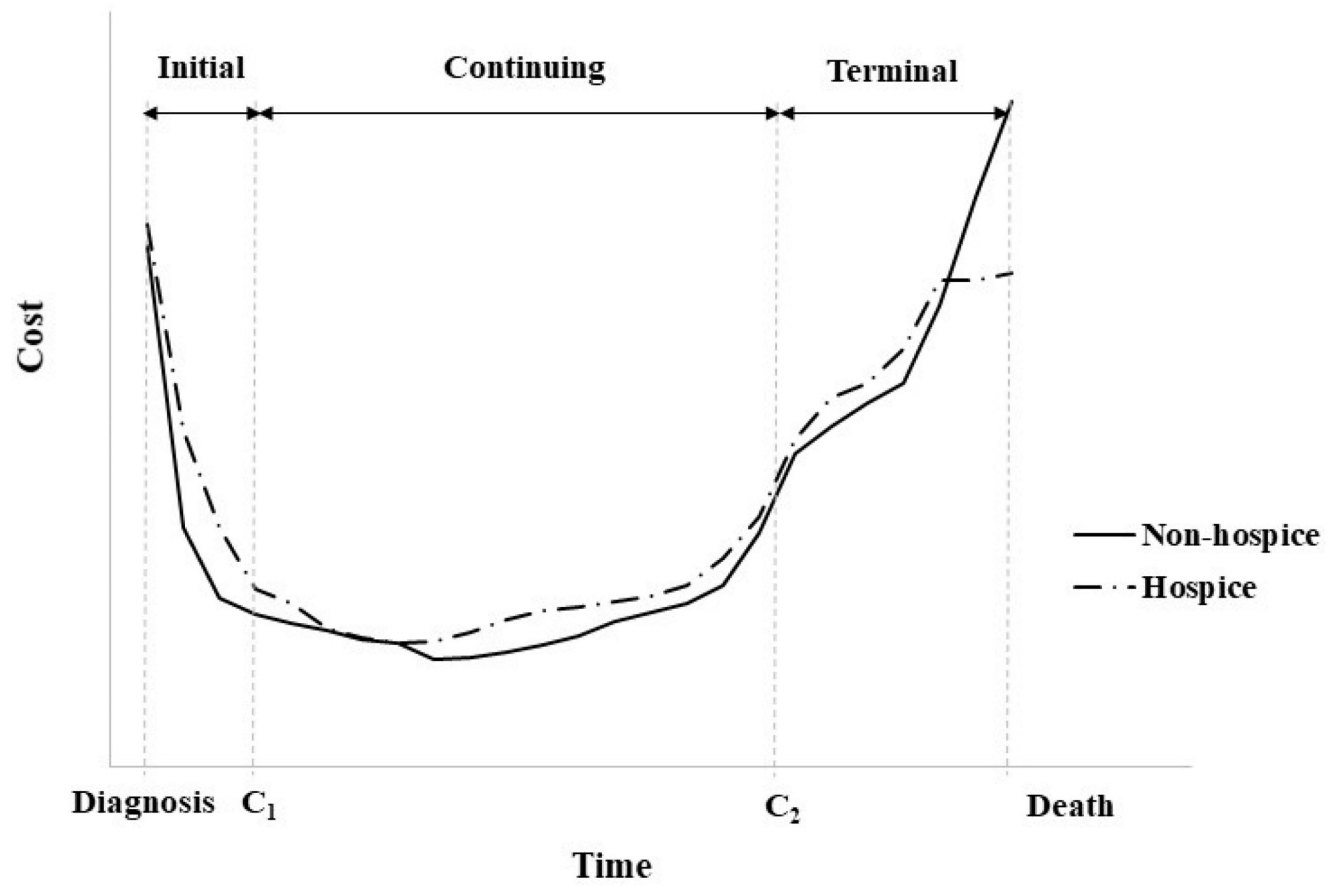
Cost trajectory for an individual patient with cancer.

In Korea, hospice and palliative care services are provided for terminal-stage patients with cancer, and 22.9% of the patients who died of cancer received this service in 2018 18. Hospitalized hospice and palliative care services started from an average of 29.3 days before death, and a flat rate of approximately $300 per hospital stay is applied. For this reason, different medical costs may apply towards their end of life to the patients who received hospice and palliative care services compared to those who died of cancer and did not receive the services (Figure 2). Analysis of the effects of hospitalized Hospice & palliative care business in Korea has reported that patients with cancer who use the services pay 40.3% less cost per day who opt for hospice care than those who do not 19.

The direct cost calculation in this study was individually tracked by converting into daily medical cost in the case of hospitalization and combining outpatient costs; this is all medical expenses related to cancer mentioned earlier. In addition, 1100 KRW was converted to one dollar by applying the exchange rate of 2021.

### Statistical analysis

The non-parameter smoothed regression model can capture both linear and non-linear terms and are defined for the response variable 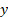 and predictor variable 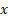 as follows:



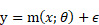



where 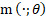 is the smoothed function with parameter 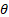; 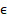 is the independent error with mean 0 and scale parameter 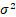 20. This function, also called locally weighted scatterplot smoothing, is robust to outliers and prevents being greatly affected by extreme observation.

Polynomial regression is suitable for non-linear functions using a polynomial term (e.g., 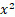, 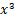), but generally does not include more than five order terms. The smoothing spline, which is an extension of it, fits each polynomial regression or step function within *K* categories for 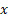 and smoothly connects at *K*-1 knots.

The change point regression (continuous piecewise linear regression) used in this study considers that the means of observations is shifted 21. The non-linear function is divided into several linear segments by unknown change points, and the direct medical costs during the cancer survival period can be set to two change points, as shown in Figure 2. The three linear segment models with two unknown change points are as follows:



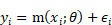





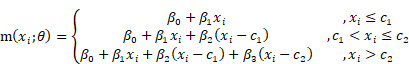



where, 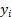, 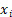, and 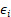 are the *i*th observation and error, respectively, and 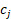 (*j* =1, 2) is the change point. The vector of parameter 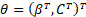 includes the vector of regression coefficients 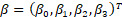 and vector of change point 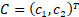. For the cost 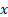, the initial phase is 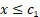, the continuing phase is 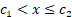, and the terminal phase is 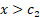. In addition, the slopes in each segment are 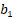, 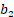, 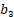 of parameters 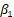, 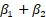, 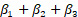. The parameter 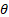 can be estimated by the non-linear least squares method through numerical optimization 22.

## Results

### Subject characteristics

The cancer death rate in patients who died within two years was the highest for pancreas cancer at 65.1% and the lowest for breast cancer at 23.6% (Table 1). However, if the fixed initial and terminal phases defined in previous studies were used, a large number of subjects would be eliminated. The cancer survival time for each type of cancer tended to be shorter in the 75^th^ quartile, comparing the median for cancer deaths in patients who used hospice and palliative care services to that of patients who did not use services. The characteristics of the study subjects according to the cancer survival time group are shown in supplementary data (Table S1). In the final year of life, the Charlson Comorbidity Index (CCI) was distributed at 8.8 to 10.6; however, the shorter the cancer survival time, the higher the score in the year before onset. These results suggest that cancer deaths with a short cancer survival time are more likely observed in patients with serious cases. No significant difference was observed in the distribution of sex, age, medical institution, region, and household income at the end of life according to the cancer survival time grouping.

**Figure 3:**
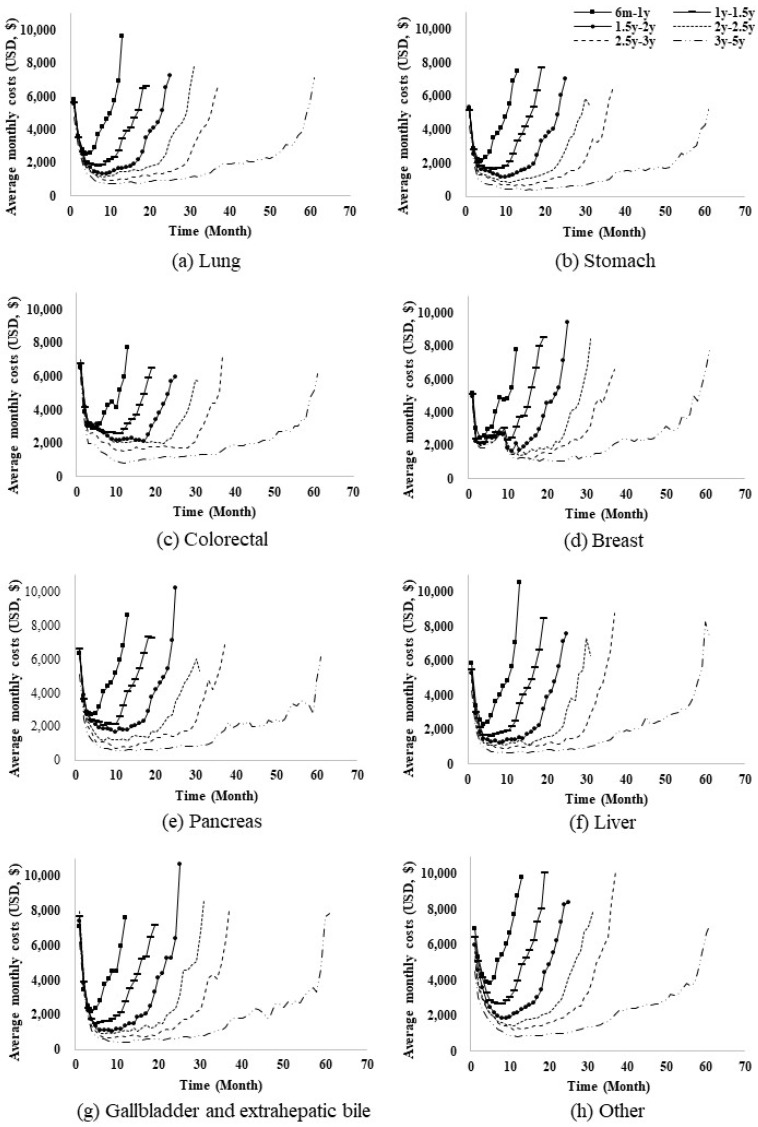
Patterns of direct cost by survival cancer time.

The pattern of the average monthly cost exhibited a U-shape, and the width was slightly different when the survival time was divided by 6 months (Figure 3). With increased cancer survival time, the flat middle period became longer and the monthly cost in the y-axis tended to decrease. Additionally, in a group with a cancer survival time of 5 years or more that also includes a survival of up to 12 years, the pattern of costs remained the same (data not shown).

### Change point estimation

The change point regression was fitted for dividing the cancer survival time by month, and regression coefficients and two change points were estimated (Table 2). 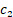, which is a terminal phase criterion, is related to the death time, and the mean of the estimates within each cancer survival time group is presented. In the cancer deaths within five years, the estimated value of 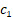 was 1.1-2.8 for seven cancer types. The difference between the death time and the estimated value of 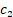 was 1.4-3.9. For patients with cancer who survived more than 5 and less than 12 years, the estimated change points were 2.3-4.8 and 2.9-4.7, respectively. Overall, as the cancer survival time increased, the initial and terminal phases tended to get longer. This indicated that the initial phase was appropriate within approximately three months after cancer diagnosis for short-term survivors and five months for long-term survivors. The terminal phase also seems reasonable to consider the final three or five months of life. The results suggest that the initial and terminal periods should be more precise than those discussed in similar previous studies exploring the cost of cancer care.

The estimated slope in the three segments represented the pattern of direct costs for cancer care. The negative slopes at the initial phase indicated that approximately $2,000 per month decreased after the month of cancer diagnosis, especially approximately $3,000 for gallbladder and extrahepatic bile cancer. The positive slopes at the terminal phase showed an increase of approximately $1,000 per month before death and up to $3,400 in the case of breast cancer patients who survived for 6 to 12 months. In addition, a modest increase or decrease was observed in the cost of cancer care in the continuing phase.

### Cost of cancer care according to the survival time

Three phases were newly defined, considering the estimated change points. The initial and terminal phases were similar at less than five months, regardless of the survival cancer time. The continuing phase, which is the middle period, varied according to the survival cancer time. Table 3 shows a summary of the direct costs of cancer care for each phase by cancer type and survival time groups in Korea. In the initial and terminal phases, the mean cost remained similar even if the survival time was shortened, but the mean cost tended to be high as the survival time shortened in the continuing phase. In the initial phase, when examination, surgery, and treatments were performed, elevated monthly costs were incurred for gallbladder and extrahepatic bile, colorectal, and pancreas cancers ($4,067-$7,431). The gap decreased as long-term survival continued. The condition of cancer deaths in the terminal phase was similarly serious, as evident in the medical cost. The mean monthly cost varied from $3,127 to $6,114 for seven cancer types in Korea, and the cost was the highest for breast cancer. This amount was calculated without distinguishing whether the patients used hospice or palliative care services.

Another point that should be focused on in the results of the study is that the cost of cancer care in the continuing phase increased with short-term survival. The highest increase was for pancreas cancer, with a monthly cost of $316 for more than five years; $943 for 3-5 years; $1541 for 2-3 years; $2,368 for 1-2 years; and $2,932 for 0.5-1 year. In the case of breast cancer, the costs for the same time periods were $1,062; $1,607; $2,096; $2,389; and $2,952; respectively. Studies using the claims data without information about the cancer stage may be difficult, and the cost of care in the continuing phase can be a good correlation to the severity of the condition. Patients who had shorter cancer survival times indicated that the cancer stage was likely to be high, and they paid more in the continuing phase.

## Discussion

This study demonstrated that by refining the definition of the initial and terminal phases, the average cost in the initial and terminal phases was shown to increase, and the cost in the continuing phase decreased. Therefore, for the calculation of medical costs by phase post-diagnosis, initial and terminal phases should be set more precisely than that defined in similar studies, considering the variations in cancer care costs. Nevertheless, these average costs may differ by country, but the pattern of change in medical costs from diagnosis to death of patients with cancer is similar; therefore, all change points can be applied in common.

Since the source is claims data, it contains all tests, treatments, and procedures, but unfortunately, the disease stage is unknown. Therefore, the severity of cancer in our study was classified according to the cancer survival time, and this could be a good alternative to the disease stage. Direct costs in the continuing phase tended to increase step by step as the cancer survival time became shorter. This indicates that if the cost of this period is properly classified and used for analysis, it can be an alternative representing the condition of patients with cancer.

In certain instances, the concept of survivors presents a unique challenge, where there may not be a clearly defined endpoint for cancer survival. Moreover, cancer-and non-cancer-related services may not be clearly distinguished, posing additional challenges. Nevertheless, our data targeted cancer deaths, thus potentially reducing various errors. In addition, our study meticulously observed all medical costs from cancer diagnosis to death and their patterns were identified. Furthermore, we constructed costs for reimbursement items, and our analysis specifically targeted clusters receiving similar care, delineated by the year of cancer death of the study subjects.

The size of medical costs at the end of life of patients with cancer who received hospice and palliative care services slightly differed from those who did not receive the services. Nevertheless, the change point for the terminal phase was not significantly affected as it became apparent when the average of the costs increased sharply. It has been estimated that the cost of hospice and palliative care services is approximately $300 per day in Korea; therefore, the medical cost at the end of life stops increasing and remains flat. Nevertheless, if the use of hospice and palliative care services before death were considered, the absolute value of the slope in the terminal phase would likely be lower for service users than that for those who did not use them. However, as our primary focus was on the costs of patients with all cancer types, we did not explore this aspect further.

It is important to acknowledge the limitations of the continuous piecewise linear regression used in the analysis, as it only considers costs as a single observation and does not account for factors like age, which may affect medical costs. Future studies should consider more complex models that can address such factors. Furthermore, in cases where the shape of the cost curve during cancer survival time varies due to healthcare system heterogeneity, adjusting the number of change points is warranted. Additionally, investigating the cost analysis according to the cancer stage in patients within the three newly defined phases would be a potential focus for future research.

## Conclusions

In this study, we scrutinized the pattern of direct costs of cancer care during the survival period for various cancer types. By estimating unknown change points, we established three distinct phases of care costs: initial, continuing, and terminal. Our change point regression analysis revealed that with an increase in cancer survival time, the initial and terminal phases became longer, reinforcing the validity of defining the initial phase as approximately three to five months after diagnosis and the terminal phase as the final three to five months before death. These findings suggest that the initial and terminal periods should be narrower than those previously considered in similar cost-of-cancer studies. Our study significantly contributes to a more comprehensive understanding of cancer care costs, emphasizing the importance of redefining these phases to ensure more accurate estimations.

## Supplementary Material

Supplementary table.

## Figures and Tables

**Table 1:** Survival time for deaths of patients with cancer who survived for more than six months

Type		N	% H	Survival time (Month)					Cancer deaths (N)				
				Q1	Q2	Q3	Mean		6 m-1 y	1-2 y	2-3 y	3-5 y	> 5 y
Lung													
	Non-H	14,560		11	22	51	37		3,985	3,812	1,851	1,862	3,050
									(27.4%)	(26.2%)	(12.7%)	(12.8%)	(20.9%)
	H	3,063	17.4%	12	22	45	35		774	864	444	439	542
									(25.3%)	(28.2%)	(14.5%)	(14.3%)	(17.7%)
Stomach													
	Non-H	8,155		13	26	56	39		1,808	2,018	1,116	1,352	1,861
									(22.2%)	(24.7%)	(13.7%)	(16.6%)	(22.8%)
	H	1,922	19.1%	13	23	46	35		449	538	317	277	341
									(23.4%)	(28.0%)	(16.5%)	(14.4%)	(17.7%)
Colorectal													
	Non-H	8,342		16	30	58	41		1,361	2,051	1,318	1,628	1,984
									(16.3%)	(24.6%)	(15.8%)	(19.5%)	(23.8%)
	H	2,125	20.3%	17	30	50	38		296	549	427	472	381
									(13.9%)	(25.8%)	(20.1%)	(22.2%)	(17.9%)
Breast													
	Non-H	2,539		24	46	79	54		206	411	351	598	973
									(8.1%)	(16.2%)	(13.8%)	(23.6%)	(38.3%)
	H	657	20.6%	27	50	80	57		32	104	89	170	262
									(4.9%)	(15.8%)	(13.5%)	(25.9%)	(39.9%)
Pancreas													
	Non-H	3,688		10	16	39	32		1,295	1,055	368	326	644
									(35.1%)	(28.6%)	(10.0%)	(8.8%)	(17.5%)
	H	1,366	27.0%	10	15	30	29		507	433	117	113	196
									(37.1%)	(31.7%)	(8.6%)	(8.3%)	(14.3%)
Liver													
	Non-H	9,761		14	32	68	45		1,922	2,075	1,255	1,663	2,846
									(19.7%)	(21.3%)	(12.9%)	(17.0%)	(29.2%)
	H	1,711	14.9%	13	28	61	40		396	385	210	287	433
									(23.1%)	(22.5%)	(12.3%)	(16.8%)	(25.3%)
Gallbladder and extrahepatic bile													
	Non-H	3,363		11	20	43	34		985	943	432	407	596
									(29.3%)	(28.0%)	(12.8%)	(12.1%)	(17.7%)
	H	871	20.6%	11	19	38	31		251	282	113	97	128
									(28.8%)	(32.4%)	(13.0%)	(11.1%)	(14.7%)
Other													
	Non-H	24,293		15	31	66	44		4,528	5,468	3,255	4,175	6,867
									(18.6%)	(22.5%)	(13.4%)	(17.2%)	(28.3%)
	H	4,533	15.7%	15	30	60	42		805	1,142	646	795	1,145
									(17.8%)	(25.2%)	(14.3%)	(17.5%)	(25.3%)

H: Hospice and palliative care service, Q1: 1^st^ quartile, Q2: Median, Q3: 3^rd^ quartile.

**Table 2:** Estimation for regression coefficients and two change points

Type		6 m-1 y			1-2 y			2-3 y			3-5 y			> 5y	
		Est	StdE		Est	StdE		Est	StdE		Est	StdE		Est	StdE
Lung															
	Intercept	5794.0	142.5		5545.7	162.0		5372.2	195.7		4645.7	226.9		2763.6	235.0
	b1	-2262.0	201.6		-1933.6	203.2		-2023.5	236.6		-1634.6	215.6		-1198.3	245.9
	b2	-52.5	77.5		26.6	13.2		27.6	5.8		21.1	3.3		8.0	1.2
	b3	1972.7	98.2		1786.6	114.3		1744.2	133.8		1932.1	167.4		1943.7	156.7
	c1	1.4	0.1		2.1	0.2		2.2	0.2		2.8	0.2		4.8	0.6
	death - c2	2.2	0.1		2.4	0.1		2.7	0.2		2.6	0.2		3.2	0.2
Stomach															
	Intercept	5203.6	175.8		5229.7	187.1		5101.9	209.2		4224.3	228.0		2767.2	252.8
	b1	-2338.9	248.5		-2572.7	264.6		-2614.4	295.8		-2198.9	299.8		-1512.0	303.0
	b2	51.7	99.5		33.0	15.0		30.3	5.8		17.2	3.0		6.2	1.1
	b3	1734.1	120.3		1617.2	119.9		1716.5	144.1		1495.8	141.2		1453.5	144.2
	c1	1.3	0.1		1.5	0.1		1.7	0.2		1.8	0.2		2.3	0.4
	death - c2	2.3	0.2		2.7	0.2		2.6	0.2		3.1	0.2		3.9	0.3
Colorectal															
	Intercept	6592.4	260.7		6613.2	236.9		6754.3	245.8		5941.0	256.8		3350.4	305.7
	b1	-2705.8	368.6		-2446.1	335.0		-3068.0	347.6		-2941.9	357.1		-1646.0	359.0
	b2	-15.5	139.1		-64.0	19.2		-28.1	6.8		7.7	3.3		7.7	1.4
	b3	1903.4	205.6		1598.5	162.0		1647.9	169.9		1614.5	164.6		2314.4	308.0
	c1	1.4	0.2		1.5	0.2		1.4	0.1		1.7	0.2		3.5	0.5
	death - c2	2.0	0.2		2.4	0.2		2.5	0.2		2.7	0.2		3.9	0.6
Breast															
	Intercept	5077.3	785.7		5038.6	503.9		4877.0	482.0		4438.7	440.8		3002.1	488.4
	b1	-1929.0	1019.2		-2773.5	664.0		-2348.2	666.3		-2304.1	617.0		-1553.3	604.3
	b2	355.7	310.7		33.8	39.1		-1.6	13.6		2.6	5.6		12.0	2.2
	b3	3400.3	1087.4		1900.8	383.4		1907.1	317.4		1989.9	279.7		1838.6	290.6
	c1	1.7	0.8		1.1	0.3		1.3	0.3		1.3	0.3		3.8	0.7
	death - c2	1.4	0.5		2.5	0.4		2.9	0.3		2.8	0.3		4.7	0.9
Pancreatic															
	Intercept	6310.9	208.6		6502.7	293.6		6547.9	403.0		5008.4	506.1		2411.5	384.8
	b1	-2792.9	295.1		-2743.6	386.0		-3375.7	551.2		-2459.5	614.4		-1417.2	468.6
	b2	6.6	120.9		6.5	23.1		27.4	11.9		18.5	7.1		6.9	2.0
	b3	1818.9	143.0		1884.5	204.6		1518.5	233.1		1496.5	252.9		1881.1	223.8
	c1	1.3	0.1		1.7	0.2		1.7	0.2		2.1	0.4		3.6	0.7
	death - c2	2.3	0.2		2.6	0.2		3.3	0.4		3.9	0.5		4.3	0.3
Liver															
	Intercept	5793.7	230.7		5367.0	270.9		5085.5	292.6		4255.4	329.9		2562.9	314.5
	b1	-2490.0	326.2		-2384.2	364.8		-2606.6	400.5		-2240.7	425.5		-1422.2	394.7
	b2	-33.5	128.6		40.5	21.3		29.8	8.2		23.9	4.3		10.2	1.4
	b3	1980.8	158.1		2305.0	188.3		2326.0	202.3		6652.6	335.9		2422.6	227.0
	c1	1.4	0.2		1.7	0.2		1.6	0.2		1.8	0.3		2.3	0.6
	death - c2	2.3	0.2		2.2	0.1		2.4	0.2		2.5	0.2		2.9	0.2
Gallbladder and extrahepatic bile															
	Intercept	7080.8	268.0		7508.0	304.1		7511.9	393.8		6180.7	455.7		3019.3	499.8
	b1	-3679.2	379.0		-3653.3	415.3		-3894.8	517.4		-2755.0	468.6		-1603.3	596.7
	b2	11.0	142.1		32.1	24.3		34.9	11.4		23.0	6.3		8.7	2.5
	b3	1756.1	183.9		1818.0	204.2		1984.1	248.3		2403.4	334.6		3294.7	327.8
	c1	1.3	0.1		1.7	0.2		1.8	0.2		2.3	0.3		3.2	0.7
	death - c2	2.5	0.3		2.7	0.2		2.9	0.3		2.8	0.3		4.6	0.5
Other															
	Intercept	6852.5	205.4		6060.5	180.4		5200.7	190.9		4179.7	178.9		2405.3	178.9
	b1	-1565.8	264.1		-1063.2	100.6		-1262.0	149.4		-942.4	113.6		-781.6	149.1
	b2	-59.5	121.6		102.4	22.8		53.0	6.9		33.7	3.2		14.7	1.3
	b3	2179.1	144.5		2198.5	146.1		2005.6	143.1		2008.3	138.7		1793.2	132.5
	c1	1.9	0.3		3.7	0.2		3.7	0.3		4.5	0.3		9.6	0.5
	death - c2	2.3	0.2		2.4	0.1		2.8	0.2		2.8	0.1		3.4	0.2

Est: mean within the survival time group for the estimates by month, StdE: mean within the survival time group for the approximate standard error of estimates by month

**Table 3:** Direct monthly cost of cancer care (US$)

Type		Initial phase (diagnosis- 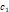 )				Continuing phase ( 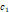 - 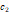 )				Terminal phase ( 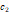 -death)		
		Mean	Q1	Q3		Mean	Q1	Q3		Mean	Q1	Q3
Lung												
	6 m-1 y	5810.6	3338.2	7628.6		2789.5	981.6	3967.5		4947.0	2312.6	6572.5
	1-2 y	5427.2	2932.4	7272.8		2074.2	870.6	2993.1		4728.7	2337.8	6389.7
	2-3 y	5128.0	2583.2	7119.4		1560.1	638.8	2195.7		4747.9	2312.9	6327.6
	3-5 y	4066.9	1601.4	5902.3		1133.9	371.8	1598.8		4525.6	2181.0	6145.0
	> 5y	2651.2	191.2	4369.4		509.5	94.2	684.3		4302.9	1963.6	5669.9
Stomach												
	6 m-1 y	5205.9	2514.8	7245.6		2364.0	750.2	3319.9		4711.4	2215.3	6411.0
	1-2 y	5226.6	2413.3	7650.9		1788.6	678.1	2552.2		4542.7	2176.4	6284.4
	2-3 y	5123.9	2378.0	7469.7		1236.6	402.6	1836.3		4351.0	2011.2	6090.7
	3-5 y	4179.0	1486.5	6494.1		752.9	185.2	1124.4		3785.0	1518.3	5337.0
	> 5y	2834.5	472.8	4531.9		397.0	84.9	532.8		3127.4	708.5	4482.4
Colorectal												
	6 m-1 y	6596.6	2878.2	9420.5		3095.9	662.5	4893.4		4810.5	2045.0	6507.2
	1-2 y	6618.3	3336.4	9332.5		2707.6	800.5	4092.9		4369.0	2033.0	5833.0
	2-3 y	6783.6	4166.4	9189.8		2168.2	745.5	3139.7		4217.9	1992.7	5802.4
	3-5 y	5907.9	3427.1	8077.8		1351.3	452.5	1993.8		4051.3	1886.5	5586.5
	> 5y	3716.8	662.8	5853.6		666.0	147.2	1007.2		3405.1	1023.2	4881.6
Breast												
	6 m-1 y	5104.0	2182.5	7241.7		2951.8	1273.3	4075.7		6114.2	2187.2	8387.8
	1-2 y	4279.4	2552.2	5764.8		2388.9	1088.8	3402.6		5471.6	2561.3	7412.2
	2-3 y	4642.4	2888.0	6117.1		2095.9	1033.8	2976.4		4902.6	2540.6	6306.3
	3-5 y	4363.3	2984.0	5761.2		1607.3	750.7	2298.1		4894.4	2427.4	6619.7
	> 5y	3189.0	1599.1	4549.8		1061.8	417.8	1476.9		4588.2	2271.9	6154.2
Pancreatic												
	6 m-1 y	6283.3	3365.7	8496.9		2931.7	1365.4	4148.4		5150.9	2839.9	6758.3
	1-2 y	6423.8	3410.4	8889.5		2368.2	1252.8	3173.6		5325.7	3026.1	6996.7
	2-3 y	6405.0	3187.3	9673.1		1541.3	736.5	2125.8		4682.3	2364.4	6225.7
	3-5 y	4738.8	1370.9	7314.7		942.6	324.3	1366.5		4276.0	2208.5	5668.7
	> 5y	2311.3	141.7	3606.3		316.3	39.1	434.2		3994.0	2047.3	5291.3
Liver												
	6 m-1 y	5795.2	2750.6	7958.8		2571.1	790.9	3509.3		4891.8	2425.5	6353.3
	1-2 y	5324.9	2287.6	7442.1		1829.2	691.8	2518.9		4893.2	2353.6	6240.6
	2-3 y	5046.1	2358.4	6896.3		1371.3	603.3	1843.0		4898.2	2346.2	6394.6
	3-5 y	4145.2	1709.1	5736.1		1014.3	447.3	1355.1		5124.1	2316.4	6386.6
	> 5y	2717.5	441.4	4010.5		602.9	203.1	818.6		4726.1	2097.2	5974.2
Gallbladder and extrahepatic bile												
	6 m-1 y	7082.3	3972.8	9466.4		2542.4	1044.9	3426.8		5061.4	2567.4	6535.1
	1-2 y	7431.3	4220.5	10157.3		1881.3	866.8	2669.0		4942.8	2560.7	6669.2
	2-3 y	7398.8	4206.7	10434.7		1386.0	709.6	1931.1		4939.2	2565.8	6572.9
	3-5 y	5385.2	2541.8	7949.5		864.7	328.2	1227.4		4926.8	2281.6	6446.5
	> 5y	2842.0	139.2	4652.7		363.8	86.9	507.6		4149.8	1994.3	5408.3
Other												
	6 m-1 y	6689.5	2374.9	9674.0		4154.7	1197.1	6073.1		6676.3	2292.3	8481.7
	1-2 y	5067.1	1588.8	7672.9		2782.6	775.7	3848.5		6341.4	2323.4	8114.5
	2-3 y	4450.1	1341.8	6512.8		1970.3	607.1	2711.5		5987.7	2237.5	7864.6
	3-5 y	3255.1	811.2	4853.9		1330.3	328.5	1870.7		5394.6	2010.2	7184.5
	> 5y	2178.5	319.8	3258.6		676.8	150.4	917.1		4608.0	1376.2	6347.8

Q1: 1^st^ quartile, Q3: 3^rd^ quartile. *1 USD $ = 1100 KRW
