# Supplementary material for: Estimation of the Three Phases by Direct Cost of Care for Non-surviving Patients with Cancer: A National Population-based Patient-level Study
Source: J Cancer. 2024 Jan 1;15(1):20–9. doi: 10.7150/jca.78491 (PMC10751664; doi:10.7150/jca.78491)
Supplement: Supplementary file 1 — Supplementary table. [file jcav15p0020s1.pdf]

Supplementary Materials

Table S1. Characteristics for deaths of patients with cancer in end of life

Characteristics	Lung N=17,623					Stomach N=10,077				
	6m-1y	1-2y	2-3y	3-5y	>5y	6m-1y	1-2y	2-3y	3-5y	>5y
Sex										
Male	3,681 (77.3%)	3,455 (73.9%)	1,578 (68.8%)	1,523 (66.2%)	2,610 (72.7%)	1,413 (62.6%)	1,696 (66.4%)	953 (66.5%)	1,130 (69.4%)	1,515 (68.8%)
Female	1,078 (22.7%)	1,221 (26.1%)	717 (31.2%)	778 (33.8%)	982 (27.3%)	844 (37.4%)	860 (33.6%)	480 (33.5%)	499 (30.6%)	687 (31.2%)
Type of medical institution										
Tertiary hospital	1,469 (30.9%)	1,349 (28.8%)	652 (28.4%)	629 (27.3%)	990 (27.6%)	556 (24.6%)	676 (26.4%)	363 (25.3%)	360 (22.1%)	460 (20.9%)
General hospital	1,722 (36.2%)	1,653 (35.4%)	828 (36.1%)	829 (36.0%)	1,352 (37.6%)	865 (38.3%)	1,002 (39.2%)	543 (37.9%)	605 (37.1%)	801 (36.4%)
Other	1,568 (32.9%)	1,674 (35.8%)	815 (35.5%)	843 (36.6%)	1,250 (34.8%)	836 (37.0%)	878 (34.4%)	527 (36.8%)	664 (40.8%)	941 (42.7%)
Region										
Capital area	1,823 (38.3%)	1,871 (40.0%)	917 (40.0%)	931 (40.5%)	1,386 (38.6%)	878 (38.9%)	1,037 (40.6%)	567 (39.6%)	587 (36.0%)	778 (35.3%)
Six metropolitan cities	1,379 (29.0%)	1,321 (28.3%)	658 (28.7%)	610 (26.5%)	993 (27.6%)	674 (29.9%)	697 (27.3%)	415 (29.0%)	448 (27.5%)	653 (29.7%)
Small cities	1,557 (32.7%)	1,484 (31.7%)	720 (31.4%)	760 (33.0%)	1,213 (33.8%)	705 (31.2%)	822 (32.2%)	451 (31.5%)	594 (36.5%)	771 (35.0%)
Household income group										
Medical care	457 (9.6%)	453 (9.7%)	239 (10.4%)	228 (9.9%)	342 (9.5%)	177 (7.8%)	282 (11.0%)	170 (11.9%)	173 (10.6%)	260 (11.8%)
1-25%	727 (15.3%)	734 (15.7%)	328 (14.3%)	322 (14.0%)	526 (14.6%)	392 (17.4%)	393 (15.4%)	207 (14.4%)	258 (15.8%)	312 (14.2%)
25-50%	776 (16.3%)	728 (15.6%)	367 (16.0%)	302 (13.1%)	473 (13.2%)	381 (16.9%)	440 (17.2%)	229 (16.0%)	251 (15.4%)	304 (13.8%)
50-75%	1,099 (23.1%)	1,047 (22.4%)	550 (24.0%)	546 (23.7%)	769 (21.4%)	496 (22.0%)	581 (22.7%)	317 (22.1%)	334 (20.5%)	454 (20.6%)
75-100%	1,700 (35.7%)	1,714 (36.7%)	811 (35.3%)	903 (39.2%)	1,482 (41.3%)	811 (35.9%)	860 (33.6%)	510 (35.6%)	613 (37.6%)	872 (39.6%)
Age mean (s.d.)	71.8 (10.4)	70.5 (10.7)	69.9 (11.0)	71.2 (10.7)	74.6 (9.6)	69.4 (14.1)	68.2 (13.8)	69.4 (13.4)	71.9 (12.9)	74.2 (11.9)
CCI in the year before onset mean (s.d.)	6.6 (3.4)	6.1 (3.2)	5.6 (3.0)	5.2 (2.8)	4.6 (2.5)	5.6 (3.0)	5.3 (2.8)	5.0 (2.6)	4.8 (2.4)	4.5 (2.3)
CCI in the final year of life mean (s.d.)	10.6 (3.5)	9.8 (3.5)	9.9 (3.5)	9.7 (3.6)	9.9 (3.7)	10.0 (3.4)	9.1 (3.4)	9.1 (3.5)	8.8 (3.4)	8.9 (3.6)

Characteristics	Colorectal N=10,467					Breast N=3,196				
	6m-1y	1-2y	2-3y	3-5y	>5y	6m-1y	1-2y	2-3y	3-5y	>5y
Sex										
Male	867 (52.3%)	1,427 (54.9%)	1,011 (57.9%)	1,286 (61.2%)	1,471 (62.2%)	3 (1.3%)	2 (0.4%)	4 (0.9%)	7 (0.9%)	9 (0.7%)
Female	790 (47.7%)	1,173 (45.1%)	734 (42.1%)	814 (38.8%)	894 (37.8%)	235 (98.7%)	513 (99.6%)	436 (99.1%)	761 (99.1%)	1,226 (99.3%)
Type of medical institution										
Tertiary hospital	374 (22.6%)	613 (23.6%)	394 (22.6%)	516 (24.6%)	526 (22.2%)	78 (32.8%)	179 (34.8%)	140 (31.8%)	248 (32.3%)	413 (33.4%)
General hospital	618 (37.3%)	973 (37.4%)	691 (39.6%)	771 (36.7%)	840 (35.5%)	68 (28.6%)	166 (32.2%)	154 (35.0%)	309 (40.2%)	447 (36.2%)
Other	665 (40.1%)	1,014 (39.0%)	660 (37.8%)	813 (38.7%)	999 (42.2%)	92 (38.7%)	170 (33.0%)	146 (33.2%)	211 (27.5%)	375 (30.4%)
Region										
Capital area	655 (39.5%)	1,049 (40.3%)	699 (40.1%)	868 (41.3%)	973 (41.1%)	124 (52.1%)	253 (49.1%)	211 (48.0%)	367 (47.8%)	580 (47.0%)
Six metropolitan cities	485 (29.3%)	724 (27.8%)	493 (28.3%)	602 (28.7%)	671 (28.4%)	51 (21.4%)	138 (26.8%)	131 (29.8%)	221 (28.8%)	353 (28.6%)
Small cities	517 (31.2%)	827 (31.8%)	553 (31.7%)	630 (30.0%)	721 (30.5%)	63 (26.5%)	124 (24.1%)	98 (22.3%)	180 (23.4%)	302 (24.5%)
Household income group										
Medical care	185 (11.2%)	312 (12.0%)	204 (11.7%)	255 (12.1%)	271 (11.5%)	37 (15.5%)	72 (14.0%)	51 (11.6%)	88 (11.5%)	175 (14.2%)
1-25%	278 (16.8%)	386 (14.8%)	306 (17.5%)	322 (15.3%)	358 (15.1%)	41 (17.2%)	78 (15.1%)	72 (16.4%)	118 (15.4%)	176 (14.3%)
25-50%	230 (13.9%)	426 (16.4%)	267 (15.3%)	336 (16.0%)	334 (14.1%)	44 (18.5%)	95 (18.4%)	82 (18.6%)	132 (17.2%)	168 (13.6%)
50-75%	345 (20.8%)	540 (20.8%)	374 (21.4%)	427 (20.3%)	486 (20.5%)	42 (17.6%)	121 (23.5%)	107 (24.3%)	179 (23.3%)	316 (25.6%)
75-100%	619 (37.4%)	936 (36.0%)	594 (34.0%)	760 (36.2%)	916 (38.7%)	74 (31.1%)	149 (28.9%)	128 (29.1%)	251 (32.7%)	400 (32.4%)
Age mean (s.d.)	73.5 (12.5)	71.0 (13.3)	69.9 (13.0)	71.0 (12.9)	75.1 (11.3)	63.4 (15.7)	60.3 (15.3)	60.5 (14.9)	59.1 (14.0)	60.5 (13.4)
CCI in the year before onset mean (s.d.)	6.2 (3.4)	5.8 (3.2)	5.2 (3.0)	4.9 (2.8)	4.5 (2.6)	4.8 (3.0)	4.4 (2.9)	3.9 (2.5)	3.7 (2.4)	3.7 (2.5)
CCI in the final year of life mean (s.d.)	10.2 (3.5)	9.5 (3.5)	9.5 (3.3)	9.7 (3.4)	9.5 (3.6)	9.5 (3.5)	9.9 (2.8)	10.2 (2.8)	10.0 (2.8)	10.2 (2.9)

Characteristics	Pancreas N=5,054					Liver N=11,472				
	6m-1y	1-2y	2-3y	3-5y	>5y	6m-1y	1-2y	2-3y	3-5y	>5y
Sex										
Male	910 (50.5%)	791 (53.2%)	255 (52.6%)	238 (54.2%)	480 (57.1%)	1,656 (71.4%)	1,785 (72.6%)	1,092 (74.5%)	1,504 (77.1%)	2,443 (74.5%)
Female	892 (49.5%)	697 (46.8%)	230 (47.4%)	201 (45.8%)	360 (42.9%)	662 (28.6%)	675 (27.4%)	373 (25.5%)	446 (22.9%)	836 (25.5%)
Type of medical institution										
Tertiary hospital	554 (30.7%)	512 (34.4%)	139 (28.7%)	136 (31.0%)	214 (25.5%)	703 (30.3%)	779 (31.7%)	463 (31.6%)	646 (33.1%)	1,085 (33.1%)
General hospital	716 (39.7%)	602 (40.5%)	190 (39.2%)	167 (38.0%)	346 (41.2%)	952 (41.1%)	971 (39.5%)	581 (39.7%)	767 (39.3%)	1,240 (37.8%)
Other	532 (29.5%)	374 (25.1%)	156 (32.2%)	136 (31.0%)	280 (33.3%)	663 (28.6%)	710 (28.9%)	421 (28.7%)	537 (27.5%)	954 (29.1%)
Region										
Capital area	787 (43.7%)	667 (44.8%)	189 (39.0%)	187 (42.6%)	347 (41.3%)	845 (36.5%)	932 (37.9%)	540 (36.9%)	767 (39.3%)	1,324 (40.4%)
Six metropolitan cities	486 (27.0%)	397 (26.7%)	146 (30.1%)	125 (28.5%)	213 (25.4%)	703 (30.3%)	702 (28.5%)	433 (29.6%)	566 (29.0%)	883 (26.9%)
Small cities	529 (29.4%)	424 (28.5%)	150 (30.9%)	127 (28.9%)	280 (33.3%)	770 (33.2%)	826 (33.6%)	492 (33.6%)	617 (31.6%)	1,072 (32.7%)
Household income group										
Medical care	126 (7.0%)	95 (6.4%)	24 (4.9%)	37 (8.4%)	76 (9.0%)	208 (9.0%)	278 (11.3%)	159 (10.9%)	230 (11.8%)	345 (10.5%)
1-25%	279 (15.5%)	193 (13.0%)	64 (13.2%)	62 (14.1%)	107 (12.7%)	402 (17.3%)	418 (17.0%)	244 (16.7%)	293 (15.0%)	509 (15.5%)
25-50%	302 (16.8%)	244 (16.4%)	73 (15.1%)	54 (12.3%)	103 (12.3%)	415 (17.9%)	394 (16.0%)	251 (17.1%)	280 (14.4%)	478 (14.6%)
50-75%	402 (22.3%)	362 (24.3%)	114 (23.5%)	94 (21.4%)	188 (22.4%)	554 (23.9%)	559 (22.7%)	343 (23.4%)	463 (23.7%)	709 (21.6%)
75-100%	693 (38.5%)	594 (39.9%)	210 (43.3%)	192 (43.7%)	366 (43.6%)	739 (31.9%)	811 (33.0%)	468 (31.9%)	684 (35.1%)	1,238 (37.8%)
Age mean (s.d.)	69.1 (11.2)	66.8 (11.4)	67.7 (11.1)	70.4 (10.8)	74.0 (9.9)	66.6 (12.6)	67.0 (12.0)	67.0 (11.6)	67.2 (11.0)	69.9 (10.6)
CCI in the year before onset mean (s.d.)	6.2 (3.1)	5.7 (2.9)	5.2 (2.6)	4.9 (2.6)	4.4 (2.5)	6.0 (3.1)	5.8 (2.8)	5.6 (2.6)	5.5 (2.6)	5.0 (2.4)
CCI in the final year of life mean (s.d.)	10.6 (3.5)	9.9 (3.5)	9.5 (3.5)	10.2 (3.6)	10.2 (3.6)	10.3 (3.7)	9.9 (3.7)	10.1 (3.9)	10.1 (3.7)	10.2 (3.8)

Characteristics	Gallbladder and extrahepatic bile N=4,234					Other N=28,826				
	6m-1y	1-2y	2-3y	3-5y	>5y	6m-1y	1-2y	2-3y	3-5y	>5y
Sex										
Male	567 (45.9%)	619 (50.5%)	311 (57.1%)	287 (56.9%)	434 (59.9%)	3,165 (59.3%)	3,958 (59.9%)	2,265 (58.1%)	2,949 (59.3%)	4,918 (61.4%)
Female	669 (54.1%)	606 (49.5%)	234 (42.9%)	217 (43.1%)	290 (40.1%)	2,168 (40.7%)	2,652 (40.1%)	1,636 (41.9%)	2,021 (40.7%)	3,094 (38.6%)
Type of medical institution										
Tertiary hospital	340 (27.5%)	339 (27.7%)	187 (34.3%)	133 (26.4%)	187 (25.8%)	1,896 (35.6%)	2,314 (35.0%)	1,308 (33.5%)	1,622 (32.6%)	2,365 (29.5%)
General hospital	462 (37.4%)	450 (36.7%)	185 (33.9%)	200 (39.7%)	269 (37.2%)	1,654 (31.0%)	2,097 (31.7%)	1,302 (33.4%)	1,598 (32.2%)	2,577 (32.2%)
Other	434 (35.1%)	436 (35.6%)	173 (31.7%)	171 (33.9%)	268 (37.0%)	1,783 (33.4%)	2,199 (33.3%)	1,291 (33.1%)	1,750 (35.2%)	3,070 (38.3%)
Region										
Capital area	463 (37.5%)	486 (39.7%)	217 (39.8%)	199 (39.5%)	286 (39.5%)	2,341 (43.9%)	2,907 (44.0%)	1,737 (44.5%)	2,156 (43.4%)	3,365 (42.0%)
Six metropolitan cities	391 (31.6%)	364 (29.7%)	154 (28.3%)	149 (29.6%)	196 (27.1%)	1,389 (26.0%)	1,796 (27.2%)	1,057 (27.1%)	1,355 (27.3%)	2,212 (27.6%)
Small cities	382 (30.9%)	375 (30.6%)	174 (31.9%)	156 (31.0%)	242 (33.4%)	1,603 (30.1%)	1,907 (28.9%)	1,107 (28.4%)	1,459 (29.4%)	2,435 (30.4%)
Household income group										
Medical care	99 (8.0%)	105 (8.6%)	46 (8.4%)	46 (9.1%)	56 (7.7%)	479 (9.0%)	651 (9.8%)	399 (10.2%)	493 (9.9%)	830 (10.4%)
1-25%	180 (14.6%)	182 (14.9%)	64 (11.7%)	69 (13.7%)	107 (14.8%)	909 (17.0%)	995 (15.1%)	559 (14.3%)	709 (14.3%)	1,077 (13.4%)
25-50%	191 (15.5%)	167 (13.6%)	96 (17.6%)	59 (11.7%)	81 (11.2%)	877 (16.4%)	1,022 (15.5%)	576 (14.8%)	719 (14.5%)	1,035 (12.9%)
50-75%	273 (22.1%)	303 (24.7%)	103 (18.9%)	119 (23.6%)	142 (19.6%)	1,101 (20.6%)	1,518 (23.0%)	912 (23.4%)	1,082 (21.8%)	1,609 (20.1%)
75-100%	493 (39.9%)	468 (38.2%)	236 (43.3%)	211 (41.9%)	338 (46.7%)	1,967 (36.9%)	2,424 (36.7%)	1,455 (37.3%)	1,967 (39.6%)	3,461 (43.2%)
Age mean (s.d.)	73.0 (10.8)	72.2 (10.6)	71.3 (10.5)	72.8 (9.9)	75.7 (9.1)	69.8 (13.8)	67.9 (14.2)	68.3 (14.2)	69.9 (13.8)	72.9 (12.9)
CCI in the year before onset mean (s.d.)	6.2 (3.1)	5.8 (2.9)	5.4 (2.9)	5.0 (2.5)	4.4 (2.4)	5.8 (3.2)	5.3 (3.1)	5.1 (3.0)	4.9 (2.9)	4.4 (2.6)
CCI in the final year of life mean (s.d.)	10.3 (3.5)	9.5 (3.7)	9.5 (3.6)	9.4 (3.7)	9.5 (3.9)	9.7 (3.7)	9.0 (3.7)	9.1 (3.7)	9.1 (3.7)	9.1 (3.8)

CCI: Charlson Comorbidity Index.
*Number of patients and percent
**Mean and standard deviation (s.d.) for age and CCI
